# Synergistic effect of asciminib with reduced doses of ponatinib in human Ph + myeloid leukemia with the T315M mutation

**DOI:** 10.1007/s12185-025-03981-7

**Published:** 2025-04-10

**Authors:** Thao Nguyen, Daisuke Harama, Minori Tamai, Keiko Kagami, Chiaki Komatsu, Shin Kasai, Koshi Akahane, Kumiko Goi, Takeshi Inukai

**Affiliations:** Global Leukemia Cell-Line Assembly Network and Department of Pediatrics, School of Medicine, University of Yamanashi, 1110 Shimokato, Chuo, Yamanashi 409 - 3898 Japan

**Keywords:** Philadelphia chromosome-positive leukemia, Tyrosine kinase inhibitor resistance, T315M mutation, CRISPR/Cas9, Synergistic effect

## Abstract

In Philadelphia chromosome-positive (Ph +) leukemia, substitution of threonine at the 315 position of BCR::ABL1 with isoleucine (T315I) induces severe resistance to tyrosine kinase inhibitors (TKIs). Of clinical importance, the substitution of the baseline T315I mutation by methionine (I315M) was reported in a Ph + leukemia patient treated with ponatinib. The resultant T315M mutation induces severe TKI-resistance in a murine Ba/F3 model. Asciminib, an allosteric inhibitor of BCR::ABL1, is reportedly active in ponatinib-resistant patients with the T315I mutation. Although asciminib alone is not active in a murine Ba/F3 model with the T315M mutation, asciminib and ponatinib show synergistic activities. In the present study, we introduced the T315M mutation into the intrinsic *BCR::ABL1* gene of two Ph + myeloid and one Ph + lymphoid leukemia cell lines using the CRISPR/Cas9 system to directly verify the utility of the combined asciminib and ponatinib in human models. All three T315M-acquired sublines were more resistant to TKIs including ponatinib than T315I-acquired sublines. Notably, asciminib exhibited a stronger synergistic effect with reduced doses of ponatinib in the T315M-acquired sublines of two myeloid cell lines, but not in the lymphoid cell line. This indicates that the combination of ponatinib and asciminib may have a clinical utility in human Ph + myeloid leukemia.

## Introduction

Tyrosine kinase inhibitors (TKIs) have dramatically improved the prognosis of patients with chronic myeloid leukemia (CML) [1] and Philadelphia chromosome-positive (Ph +) acute lymphoblastic leukemia (ALL) [2]. Despite its clinical utility, TKI resistance occurs in some patients due to the point mutation of the *BCR::ABL1* gene [3]. Imatinib and second-generation TKIs bind to the ATP-binding pocket of BCR::ABL1 and block the aberrant kinase activity of BCR::ABL1. Among residues constituting the ATP-binding pocket of BCR::ABL1, threonine at the 315 position plays a critical role in stabilizing interactions between BCR::ABL1 and TKIs [3]. When threonine at the 315 position is replaced with isoleucine (T315I mutation), the interaction between the ATP-binding pocket and TKIs is massively abrogated [4]. As a result, the T315I mutation of BCR::ABL1, referred to as the “gatekeeper” mutation, induces severe resistance to imatinib and second-generation TKIs.

Ponatinib is a high-affinity pan-BCR::ABL1 kinase inhibitor with unique activity against BCR::ABL1 with the T315I mutation [5]. The carbon–carbon triple bond linkage of ponatinib allows effective hydrophobic contact with the side chain of the T315I gatekeeper mutation [5]. The anti-leukemic activity of ponatinib has been confirmed in clinical trials, including the patients with the T315I mutation [6]. Of clinical importance, baseline T315I mutation is reportedly substituted by the I315M mutation along with disease progression in one Ph + ALL patient treated with ponatinib [7]. In this case with a baseline T315I mutation, a complete cytogenetic response was observed by ponatinib (45 mg/day). However, emergence of a ponatinib-resistant clone with a change of I315 to I315M was observed seven months later through a single nucleotide change (ATT to ATG). Consistently, in a murine Ba/F3 model of the BCR::ABL1 with the T315I mutation, development of the I315M mutation was confirmed in 1/141 (0.7%) of the ponatinib-resistant clones treated with 40 nM of ponatinib [5], and in 1/23 (5%), 6/27 (22%), and 1/6 (17%) of the rebastinib-resistant clones treated with 250 nM, 500 nM, and 750 nM of rebastinib, respectively [8]. Molecular dynamics simulation was reported to reveal a deep penetration of M315 into the ponatinib site of the ATP pocket, with disrupted structural adjustments in the other residues [7]. However, the prevalence of the I315M mutation as the ponatinib-resistant mechanism in the TKI-treated CML cases with a baseline T315I mutation remains unclear, since previous studies mainly focused on the acquisition of T315I-inclusive compound mutations but not the replacement of T315I mutation [9, 10]. Notably, in a murine Ba/F3 model, the resultant T315M mutation of BCR::ABL1 was confirmed to show the highest resistance to second- and third-generation TKIs, including ponatinib [7], as the E255 V/T315I mutation, which is a representative T315I-inclusive compound mutation with the highest resistance to all TKIs including ponatinib [7]. Thus, since T315M is the most influential single mutation, the establishment of a human Ph + leukemia model with the T315M mutation could be useful to develop a novel therapeutic strategy for overcoming ponatinib-resistance.

Asciminib, an allosteric inhibitor of BCR::ABL1, binds to the myristoyl-binding site [11, 12]. The myristoyl pocket of ABL1 is normally occupied by the myristoylated N-terminal of ABL1, which is an allosteric negative regulatory element of kinase activity. Notably, the myristoylated N-terminal of ABL1 is lost in BCR::ABL1 as a result of fusion. Asciminib mimics myristate by binding to the myristoyl site and, subsequently, inhibits the kinase activity of BCR::ABL1 by inducing an inactive closed conformation. Accordingly, asciminib is active against BCR::ABL1, even with the T315I gatekeeper mutation [13]. Indeed, asciminib is reportedly active in heavily pretreated CML patients with TKI resistance, including ponatinib-resistant patients with the T315I mutation [14–16]. Notably, crystallography studies showed that asciminib and other TKIs can co-bind a single molecule of BCR::ABL1, and in vitro studies revealed additive effects of asciminib with TKIs [13]. In particular, although asciminib alone was not effective against BCR::ABL1 with the T315M mutation in the murine Ba/F3 model, asciminib combined with ponatinib showed synergistic activity [13]. It has been speculated that conformational changes in mutated BCR::ABL1 induced by transient ponatinib binding enable the assembly of asciminib to the myristoyl-binding pocket, which in turn further stabilizes ponatinib binding [5].

For pharmacological evaluation of the BCR::ABL1 mutation, the murine Ba/F3 system, in which wild-type or mutated cDNA of the *BCR::ABL1* fusion gene is retrovirally transduced, has been widely used as a model [4]. However, despite its utility, the murine Ba/F3 system has several limitations as a model for human Ph + leukemia. First, Ba/F3 is a murine cell system. Second, it induces overexpression of *BCR::ABL1* fusion derived from cDNA under the control of the viral promoter. Third, the introduced *BCR::ABL1* cDNA lacks introns and a majority of the 3′ untranslated region (UTR). In this context, the 3′ UTR of the *BCR::ABL1* gene reportedly plays a role in microRNA-mediated post-transcriptional regulation [17]. Moreover, alternative splicing of the *BCR::ABL1* gene is reportedly involved in TKI resistance [18].

To overcome these limitations in the murine Ba/F3 model, direct induction of the mutation into the intrinsic *BCR::ABL1* gene of human Ph + leukemia cells is an ideal model. Recently, via homologous recombination (HR) using CRISPR/Cas9 system, we introduced T315I gatekeeper mutation into the intrinsic *BCR::ABL1* fusion gene in human Ph + myeloid leukemia cell lines [19] and Ph + lymphoid leukemia cell line [20]. In the present study, using the same strategy, we successfully introduced the T315M mutation into human Ph + myeloid and lymphoid leukemia cell lines. Using these sublines, we evaluated the synergistic activities of asciminib and TKIs and confirmed the excellent synergy between ponatinib and asciminib in myeloid cell lines, but not in the lymphoid cell line.

## Materials and methods

### Cell lines

TCCS [21] and KOPM28 [22] were established from CML patients in myeloid blast crisis with the p210 BCR::ABL1, while KOPN55bi was established from a CML patient in lymphoid blast crisis with the p210 BCR::ABL1 [20, 22]. All three cell lines were confirmed to have only the *BCR::ABL1* alleles but not the *ABL1* alleles [19, 20]. All cell lines were maintained in RPMI1640 medium supplemented with 10% fetal calf serum (FCS).

### Genome editing

**Fig. 1:**
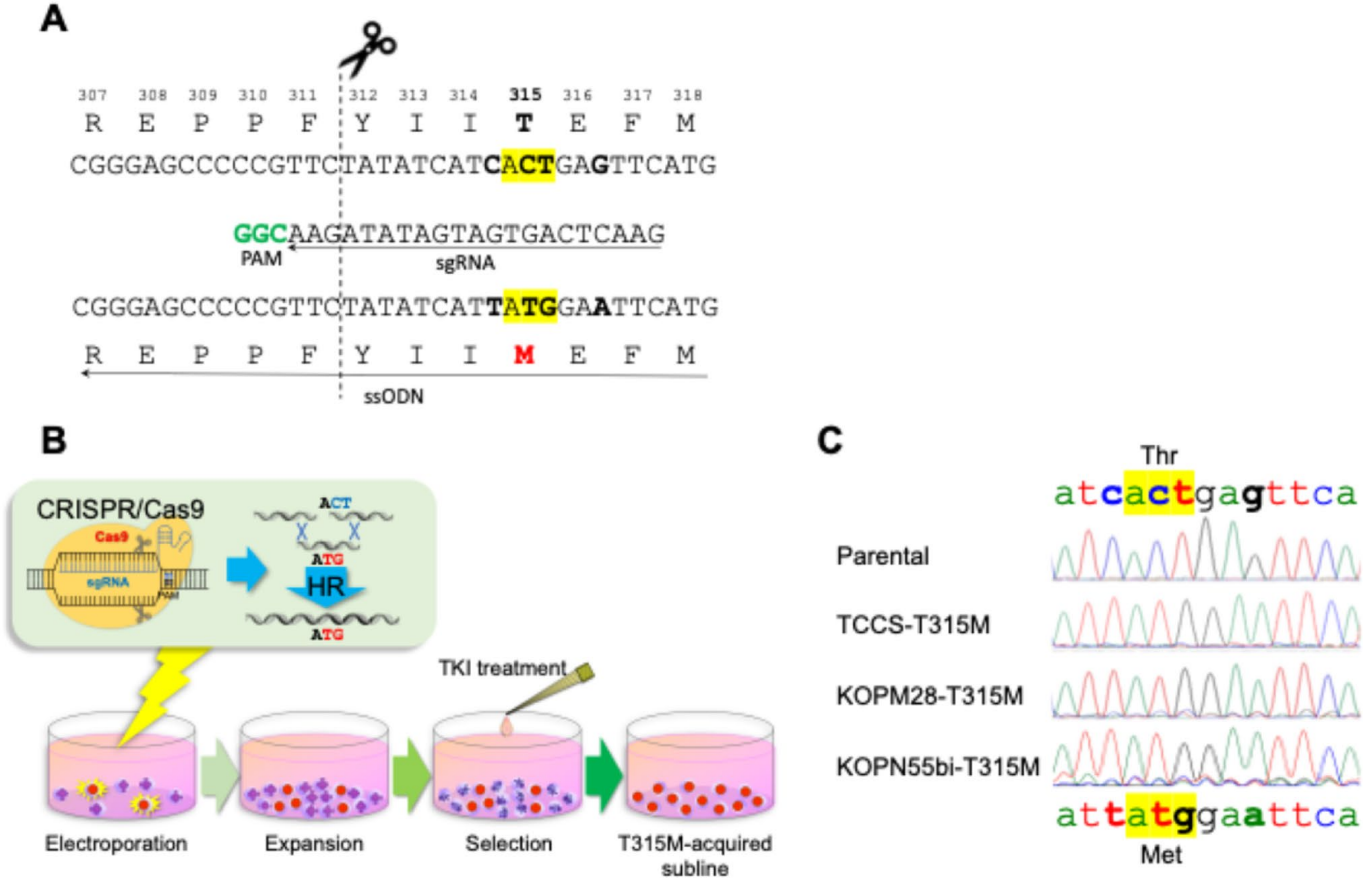
Establishment of T315M-acquired sublines in three human Ph + leukemia cell lines. **A** Schematic diagram of the sgRNA and the ssODN. The top indicates wild-type amino acid and nucleotide sequences, the middle indicates the sequence of sgRNA, and the bottom indicates the sequence of ssODN. In the sgRNA sequence, the PAM site is indicated in bold green. The scissors icon and dotted line indicate the cleave site of Cas 9 nuclease. Codon 315 is highlighted in yellow. Four mutated nucleotides are indicated in bold. **B** Transfection and selection workflow. **C** Genomic sequencing of the PCR products of the three T315M-acquired sublines. Wild-type genomic sequences are indicated at the top, and mutated genomic sequences are indicated at the bottom

The detailed protocol has been described previously [19, 20]. CRISPR guide sequences were designed using the CRISPR design tool (CRISPR DESIGN, http://crispr.mit.edu) as follow; 5′-atcactgagttcatgacctacgg- 3′ (forward sgRNA) and 5′-aactcagtgatgatatagaacgg- 3′ (reverse sgRNA) (Fig. 1A). The 102 base pair template ssODN in a non-transcribed strand (anti-sense) direction were synthesized by Integrated DNA Technologies (Coralville, IA). The sequence of ssODN in the sense direction was 5′-cgttcacctcctgccggttgcactccctcaggtagtccaggaggttcccgtaggtcatgaattccataatgatatagaacgggggctcccgggtgcagaccc- 3′. Before electroporation, cells were pre-treated with 10 nM of SCR7 (Cayman Chemical, Ann Arbor, MI), a non-homologous end-joining inhibitor, for 24 h to enhance HR efficiency [23]. Subsequently, recombinant Cas9 (Integrated DNA Technologies) nuclease and guide RNA as a ribonucleoprotein complex with template ssODN were transfected into 5 × 10^5^ of the pre-treated cells by electroporation using the Neon electroporation transfection system (ThermoFisher Scientific, Waltham, MA). The electroporated cells were transferred to a 96-well plate and cultured in the presence of 10 nM of SCR7 for 48 h and the cells were further expanded for 7 days. After a 7-day culture in the absence of TKIs, TCCS and KOPM28 were cultured in the presence of 1 μM of imatinib for 14 days, while KOPN55bi was cultured in the presence of 100 nM of dasatinib for 14 days (Fig. 1B). We obtained TKI-resistant sublines of TCCS, KOPM28, and KOPN55bi 18, 21, and 24 days after electroporation, respectively. The cells were transferred to culture flasks and expanded in the absence of TKI for further experiments.

### Direct sequencing

Genomic DNA was extracted using a PureLink Genomic DNA Mini Kit (ThermoFisher Scientific) and amplified by PCR using forward (5′-ccacacgagcacagtctcag- 3′) and reverse (5′-accttcaccaagtggttctcc- 3′). Direct sequencing of each PCR product was performed using the forward primer, and confirmed introduction of T315M and silent point mutations without a wild-type sequence (Fig. 1C).

### Cell viability assay

To determine the sensitivities to TKIs and asciminib, an alamarBlue cell viability assay (Bio-Rad Laboratories, Hercules, CA) was performed. A total of 1–5 × 10^5^ cells were plated into a 96-well flat-bottom plate in triplicate and cultured for 72 h in the presence or absence of each drug. After a 6-h additional incubation with alamarBlue, absorbance at 570 nm was monitored by a microplate spectrophotometer using 600 nm as a reference wavelength. Cell survival was calculated by expressing the ratio of the optical density of the treated wells to that of the untreated wells as a percentage. The concentration of the drug required to reduce the viability of treated cells to 50% of untreated cells was calculated, and the median of three independent assays was determined as the 50% inhibitory concentration (IC50) for each cell line.

### Western blot analyses

Cells were solubilized in lysis buffer (50 mM Tris–HCl, pH 7.5, 150 mM NaCl, 1% Nonidet P- 40, 5 mM EDTA, 0.05% NaN3, 1 mM phenylmethylsulfonyl fluoride, 100 μM sodium vanadate). The lysates were separated on an SDS–polyacrylamide gel under reducing conditions and then transferred to a nitrocellulose membrane. The membrane was incubated with anti-phospho-tyrosine (4G10; Cell Signaling Technology, Danvers, MA) and anti-alpha-Tubulin antibody (T5168; Sigma Aldrich, St. Louis, MO) at 4 °C overnight, and then with horseradish peroxidase-labeled second antibody (MBL, Nagoya, Japan) at room temperature for 1 h. The bands were developed using an enhanced chemiluminescence detection (ECL) kit (GE Healthcare, Little Chalfont, UK).

### Evaluation of synergistic effects

The synergistic effects were analyzed using SyngergyFinder software [24]. Since the ZIP model provides an improved solution for identifying true synergistic interactions with a relatively low false-positive rate [25], we chose to present ZIP scores. In the ZIP model, the combined effect of the two drugs was captured by comparing the changes in the potency of the dose–response curves between each individual drug and their combinations.

## Results

### TKI-sensitivity in the T315M-acquired sublines

**Fig. 2:**
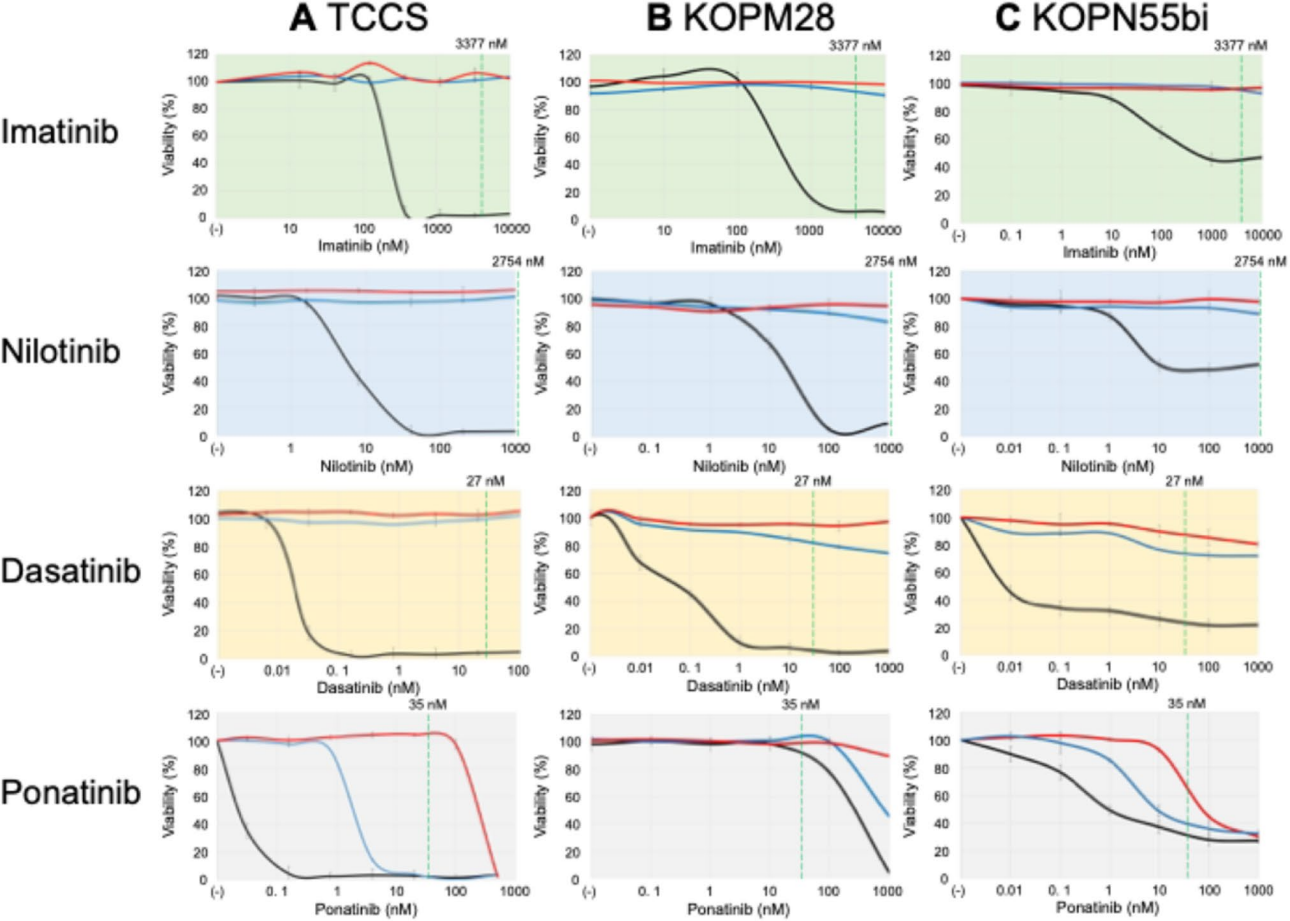
Dose–response curves of TKIs in **A** TCCS, **B** KOPM28, and **C** KOPN55bi. The black, blue, and red lines indicate dose–response curves in the parental cells, the T315I-acquired sublines, and the T315M-acquired sublines, respectively. The horizontal and vertical axes indicate the concentration of TKIs and viability, respectively. Error bars indicate standard errors in triplicated analyses. Green dotted line indicates average steady-state plasma concentration of each TKI

We evaluated the TKI sensitivities of three T315M-acquired sublines in comparison with the previously established T315I-acquired sublines [19, 20] as well as their parental cells. First, we evaluated the sensitivities to imatinib, dasatinib, and nilotinib (Fig. 2A–C). The parental cells of TCCS and KOPM28 were highly sensitive to these three TKIs. In comparison with these two myeloid cell lines, the parental cells of KOPN55bi were relatively resistant to imatinib and nilotinib. Consistent with its property as a dual Src and Abl kinase inhibitor [26], dasatinib showed higher anti-leukemic activity in the parental cells of KOPN55bi. In contrast to the parental cells, all three T315I-acquired sublines were highly resistant to all three TKIs. Moreover, consistent with the findings in the murine Ba/F3 model [7], all three T315M-acquired sublines were also highly resistant to the three TKIs.

We next evaluated the sensitivity to ponatinib (Fig. 2A–C). In three parental cells, TCCS was highly sensitive to ponatinib, while KOPM28 and KOPN55bi were moderately sensitive to ponatinib. All three T315I-acquired sublines were significantly more resistant to ponatinib compared with their parental cells. Moreover, all three T315M-acquired sublines were significantly more resistant to ponatinib than the T315I-acquired sublines. These observations indicated that the T315M mutation induces higher levels of TKI resistance than the T315I mutation in human Ph + leukemia cell lines.

### Tyrosine phosphorylation status of cellular proteins in the T315M-acquired sublines

**Fig. 3:**
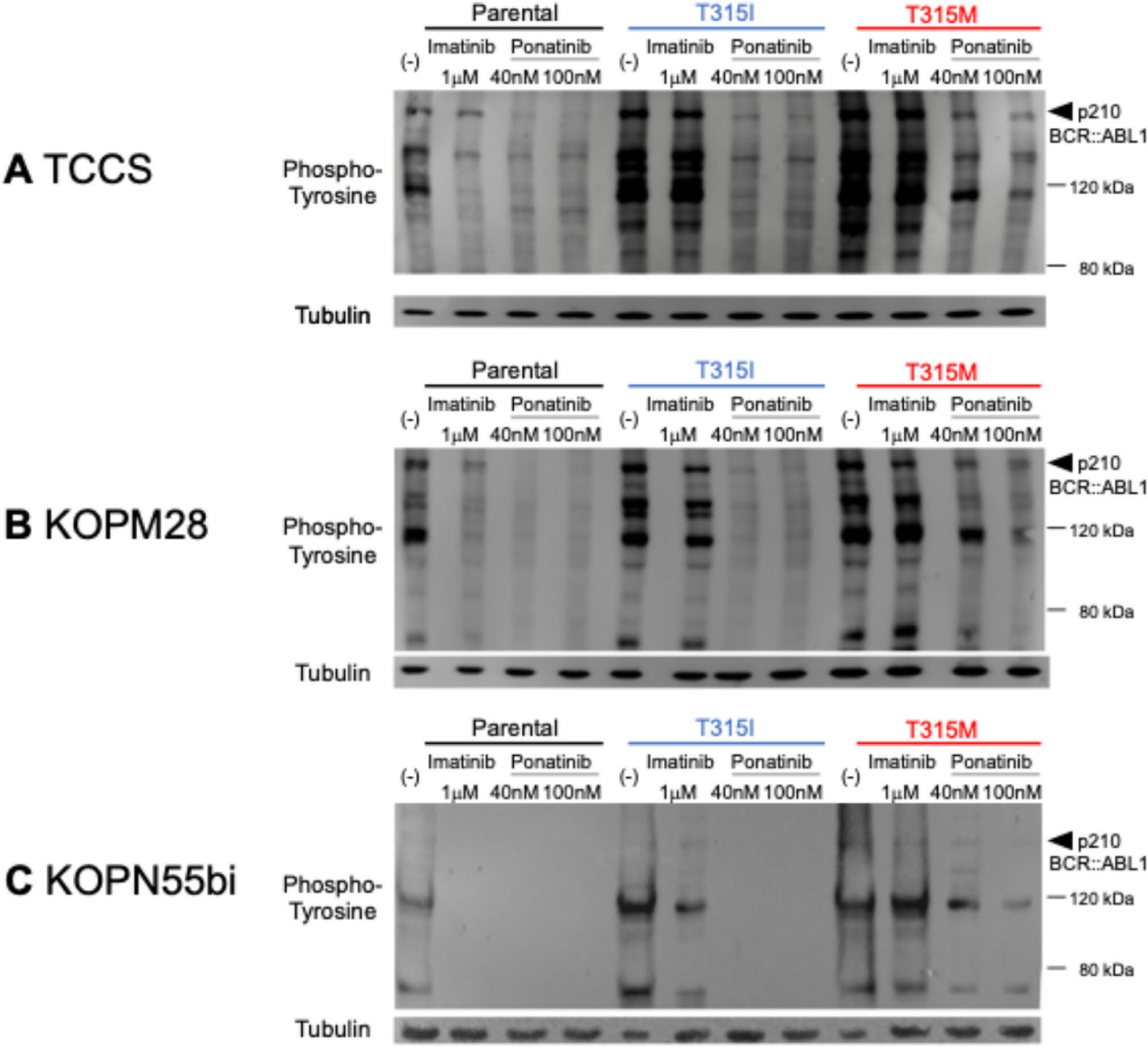
Western blot analysis of tyrosine phosphorylation in the parental cells, the T315I-acquired sublines, and the T315M-acquired sublines of **A** TCCS, **B** KOPM28, and **C** KOPN55bi. The cells were cultured in the absence or presence of imatinib at 1 µM, or ponatinib at 40 nM or 100 nM for 12 h. The top panels indicate the blots of anti-phospho-tyrosine antibody, and the bottom panels indicate the blots of anti-alpha Tubulin antibody as internal controls

We next evaluated the significance of the T315M mutation in the tyrosine phosphorylation status of cellular proteins after TKI treatment. Using anti-phospho-tyrosine antibody, phosphorylation status of cellular proteins was analyzed in the T315M and T315I-acquired sublines as well as their parental cells after treatment with 1.0 µM of imatinib or 40 nM and 100 nM of ponatinib for 12 h by Western blotting (Fig. 3A–C). In the parental cells of the three cell lines, tyrosine residues of cellular proteins were largely dephosphorylated by treatment with imatinib and ponatinib. In the three T315I-acquired sublines, tyrosine residues of cellular proteins were almost not dephosphorylated by imatinib, while tyrosine residues of cellular proteins were largely dephosphorylated by 40 nM and 100 nM of ponatinib. In three T315M-acquired sublines, tyrosine residues of cellular proteins were not dephosphorylated by imatinib. Notably, tyrosine residues of cellular proteins were almost not dephosphorylated by 40 nM of ponatinib and partially dephosphorylated by 100 nM of ponatinib. These observations indicate that the T315M mutation induces higher levels of ponatinib resistance than dose the T315I mutation in human Ph + leukemia cell lines.

### Synergistic effect of asciminib with TKIs in the T315M-acquired sublines

We investigated the combined effect of asciminib and other TKIs in three T315M-acquired sublines using the alamarBlue assay. Three T315M-acquired sublines were treated with asciminib up to 2 mM (therapeutic concentration of asciminib; 1.7–3.9 mM) [14] in combination with imatinib up to 10 mM (2.4–5.2 mM) [27], nilotinib up to 2 mM (1–2.3 mM) [28], dasatinib up to 500 nM (11.7–494 nM) [29], or ponatinib up to 200 nM (23.4–138 nM) [5, 30] for 72 h. Based on the results of the alamarBlue assay, we calculated the ZIP synergy score [25] using the SynergyFinder software [24].

**Fig. 4:**
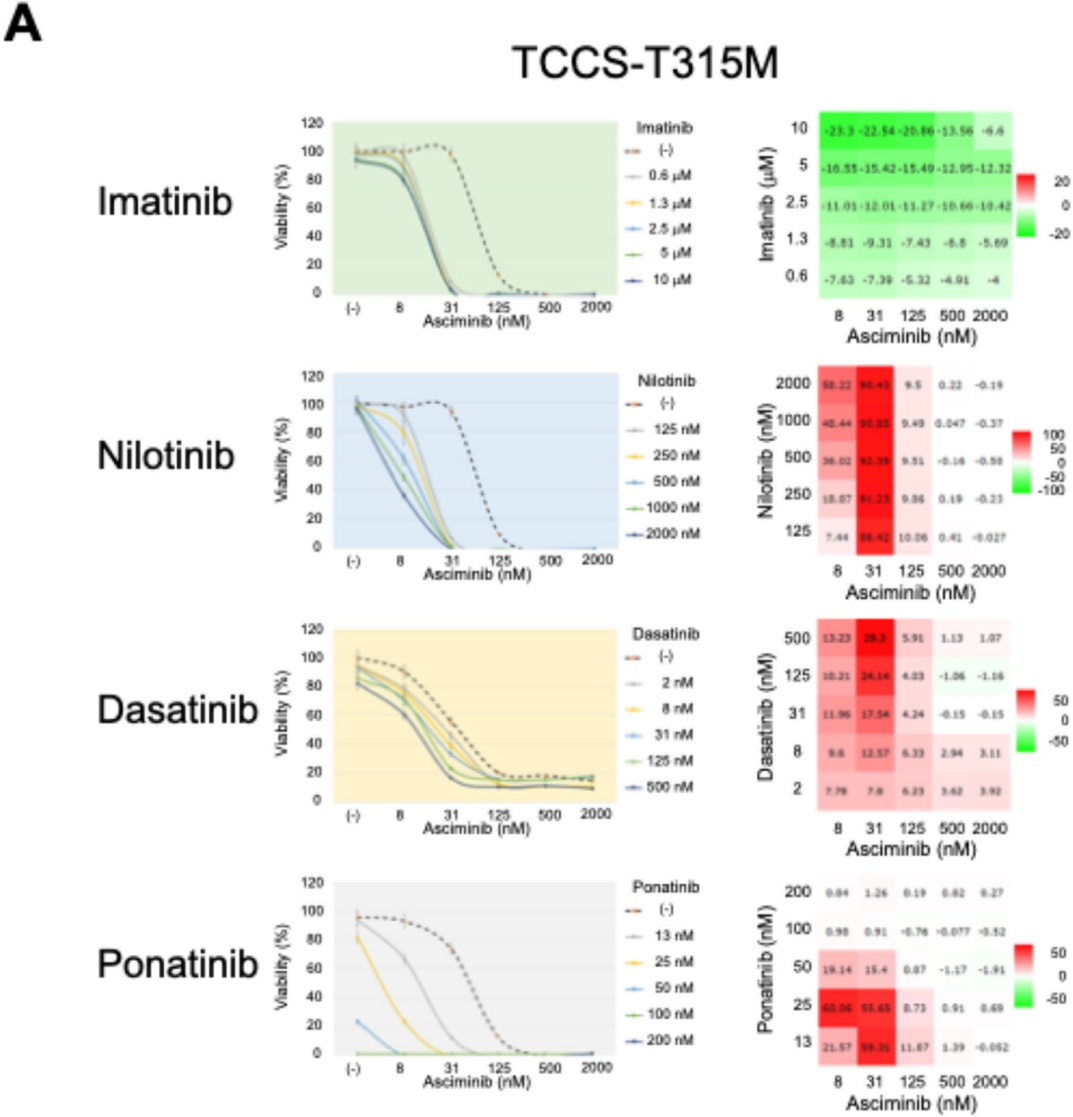

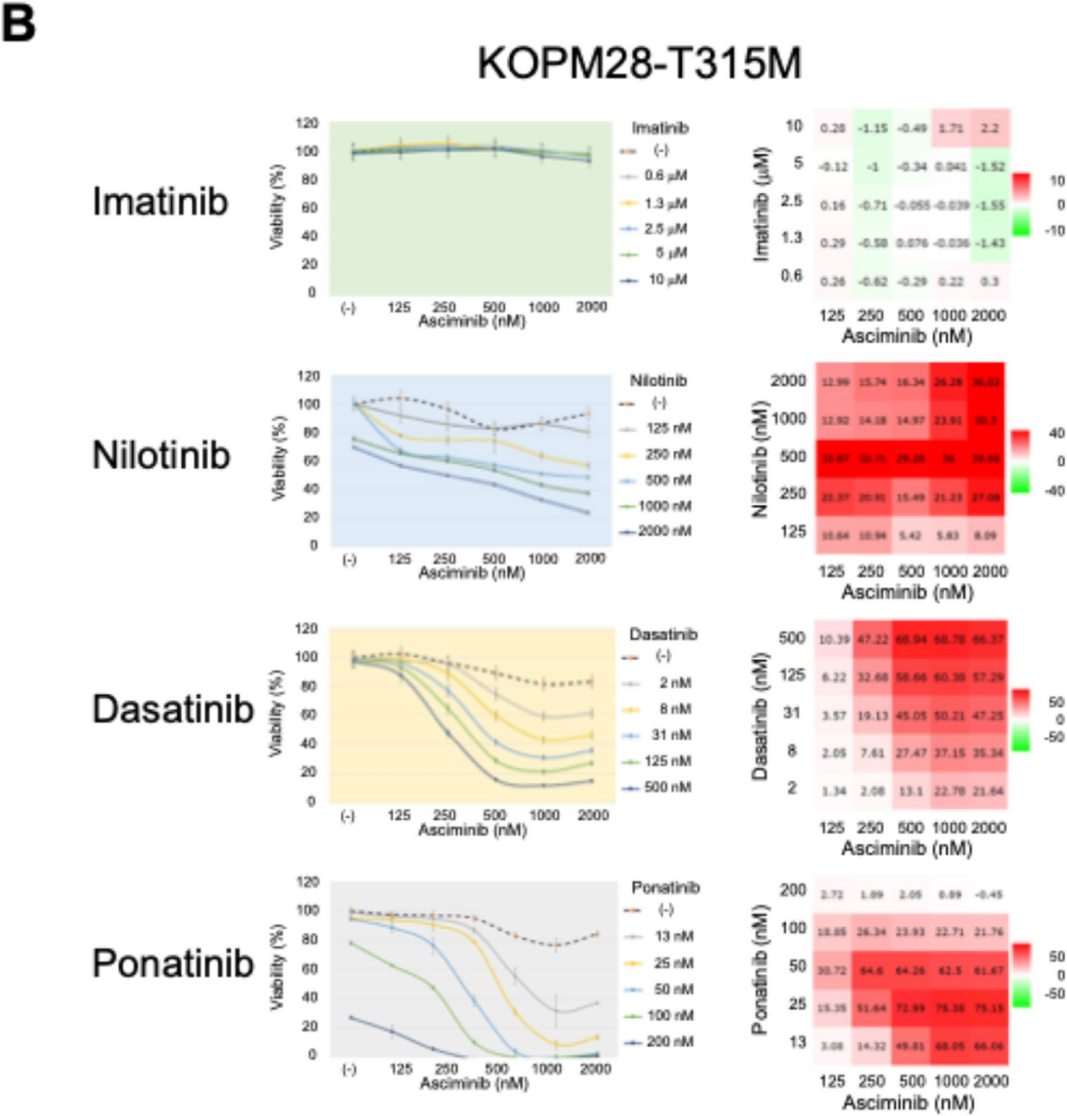

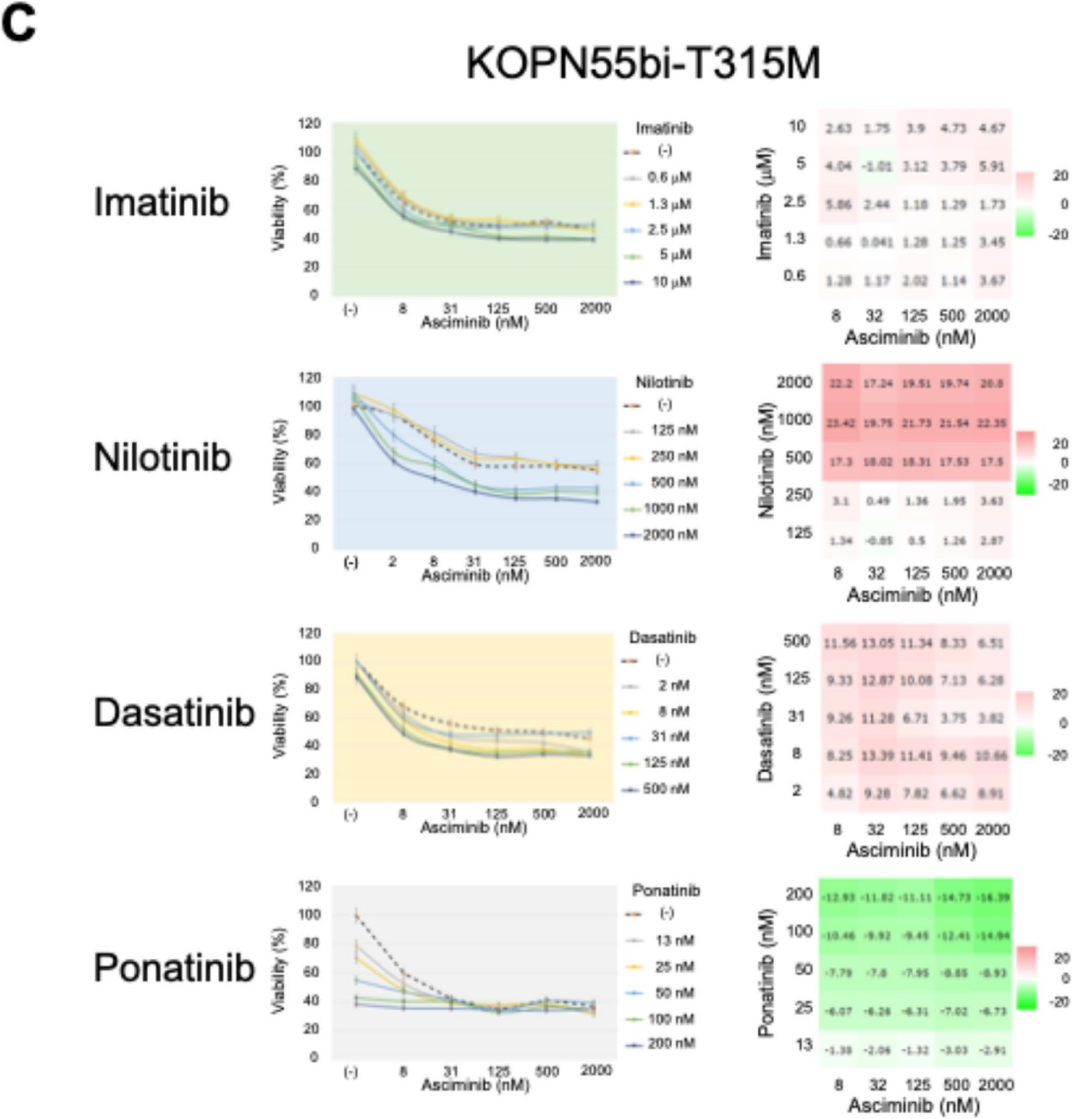
Combination effects of asciminib and other TKIs in the T315M-acquired sublines of **A** TCCS, **B** KOPM28, and **C** KOPN55bi. The cells were treated with asciminib and each TKI as a single agent or in combinations at various concentrations. The left panels indicate the dose–response curves. The horizontal and vertical axes indicate the concentrations of asciminib and cell viabilities, respectively. Concentrations of each TKI are indicated on the right side of each dose-dependent curve. Error bars indicate standard errors in triplicated analyses. The right panels indicate heat maps of ZIP synergy scores. The horizontal and vertical axes indicate the concentrations of asciminib and each TKI. Color scales of the heat map are indicated on the right side of each map

In the T315M-acquired subline of TCCS, asciminib alone showed anti-leukemic activity in a dose-dependent manner (Fig. 4A). The median IC50 value of asciminib was approximately 100 nM. Imatinib did not show any synergistic effects with asciminib, while nilotinib at 125–2000 nM showed high levels of synergistic effects (ZIP synergy score > 50) with asciminib at 31 nM. Moreover, dasatinib at 125–500 nM showed weak synergistic effects (> 20) with asciminib at 31 nM. Finally, ponatinib showed higher levels of synergistic effects when combined with asciminib. The best synergy was observed between ponatinib at 25 nM and asciminib at 8 nM (ZIP synergy score: 60), since the T315M-acquired subline of TCCS was almost completely killed by combinations between ponatinib > 50 nM and asciminib > 125 nM.

In the T315M-acquired subline of KOPM28, asciminib of up to 2 mM alone failed to show anti-leukemic activity (Fig. 4B). Although imatinib did not show any synergistic effects with asciminib, nilotinib at 250–2000 nM showed weak synergistic effects (ZIP synergy score > 20) with asciminib at 125–2000 nM. Both dasatinib at 31–500 nM and ponatinib at 13–50 nM showed high synergistic effects (> 50) with asciminib at 250–500 nM in a dose-dependent manner. The highest ZIP synergy scores (ZIP synergy scores: 68.9 and 75.3, respectively) were observed between dasatinib at 500 nM and asciminib at 500 nM and between ponatinib at 25 nM and asciminib at 1000 nM.

In the T315M-acquired subline of KOPN55bi, asciminib alone showed a moderate anti-leukemic activity in a dose-dependent manner (Fig. 4C). Imatinib did not show any synergistic effects with asciminib, while nilotinib at 50–2000 nM and dasatinib at 8–500 nM showed very weak synergistic effects (ZIP synergy score > 10) with asciminib at 8–2000 nM. Notably, in contrast to the T315M-acquired sublines of the two myeloid cell lines, ponatinib did not show any synergistic effects with asciminib in the T315M-acquired subline of KOPN55bi. These observations indicated that the combination of ponatinib and asciminib shows higher levels of synergistic effects in T315M-acquired Ph + myeloid cell lines but not in T315M-acquired Ph + lymphoid cell line.

## Discussion

In the present study, we introduced the T315M mutation into the intrinsic *BCR::ABL1* gene in three Ph + leukemia cell lines via HR using the CRISPR/Cas9 system. In the obtained TKI-resistant sublines, we confirmed the acquisition of both T315M and additional silent mutations. Thus, these three sublines acquired the T315M mutation as a result of HR. The obtained three T315M-acquired sublines were highly resistant to imatinib, nilotinib, and dasatinib. Moreover, all three T315M-acquired sublines were significantly more resistant to ponatinib than the previously established T315I-acquired sublines.

Notably, we confirmed higher levels of synergistic effects between ponatinib and asciminib in the T315M-acquired sublines of two myeloid Ph + leukemia cell lines. In ponatinib therapy, due to dose-dependent cardiovascular toxicity, elective dose reduction to 30 or 15 mg/dose is recommended [31, 32]. When treated with a reduced 15 mg/dose of ponatinib, the serum concentration at steady state was reported to be approximately 25 nM [30]. Of clinical importance, in both T315M-acquired sublines of the two Ph-positive myeloid cell lines, a strong synergistic effect (ZIP synergy score > 50) was observed at 13 nM and 25 nM of ponatinib. Thus, our observations suggested that asciminib combined with low-dose ponatinib could be effective in patients with CML or myeloid crisis with the T315M mutation [5].

For clinical application of combination therapy, confirmation of adverse effects on non-leukemic cells is crucial. Although no previous studies directly focused on this issue, there are a couple of indirect observations in several publications. First, in xenograft mouse models [13, 33], the combination did not exhibit exceptional toxicity in terms of animal weight loss. Second, there are two case reports of combination therapy. In a 52-year-old female CML case acquiring a T315I/E335G compound mutation [34], combination treatment induced a rapid hematologic response and stable disease for several months, suggesting no serious bone marrow suppression. However, the patient was finally intolerable to a combination of asciminib at 80 mg bis in die (BID) with an increased dose of ponatinib from 15 mg quaque die (QD) to 30 mg QD due to worsening of fatigue and weakness. Meanwhile, in a 75-year-old male Ph + ALL case refractory to standard therapy [35], combination treatment induced a hematologic response with normocellular marrow for three months as a bridge therapy, suggesting no serious bone marrow suppression. The patient was well tolerated to asciminib at 40 mg BID and ponatinib at 30 mg QD without any specific adverse events.

Unexpectedly, in the present study, a synergistic effect between ponatinib and asciminib was not confirmed in the T315M-acquired subline of KOPN55b. Fundamental oncogenic properties of BCR::ABL1 in Ph + lymphoid leukemia cells must be identical to those in Ph + myeloid leukemia cells. BCR::ABL1 acts as a ‘super adaptor’ via its own Src homology 2 (SH2) domain by direct interactions with multiple core components of diverse pathways implicated in the oncogenic transformation [36]. Thus, TKIs are considered to interfere with the equilibrium state of these diverse protein complexes [36, 37]. Notably, the interacting cellular proteins and their phosphorylation status are partially different between Ph + lymphoid leukemia cells and Ph + myeloid leukemia cells [38]. These differences might contribute to different responses to the combination of ponatinib and asciminib between the Ph + lymphoid leukemia cell line (KOPN55bi) and the Ph + myeloid leukemia cell lines (TCCS and KOPM28) at least in part. The other possible background for resistance to the combination of ponatinib and asciminib in the T315M-acquired subline of KOPN55b might be the disruption of the apoptotic process, which is regulated by the downstream signaling pathway of BCR::ABL1.

In a murine Ba/F3 model, which is considered to be pro-B cell line [39], asciminib and ponatinib reportedly showed synergistic activity in the T315M cells [13]. Thus, our observation in the T315M subline of KOPN55bi was potentially inconsistent with this finding in a murine Ba/F3 model. Under these circumstances, confirmation in the other Ph + lymphoid leukemia cell line(s) is required. To introduce specific mutation s via HR using the CRISPR/Cas9 system, the cellular HR pathway must be intact. However, as we already reported [20], among 16 Ph + lymphoid leukemia cell lines we had tested, 10 cell lines were sensitive to olaparib, a poly (ADP-Ribose) polymerase- 1 (PARP- 1) inhibitor, suggesting that their HR pathway is somehow disrupted. Meanwhile, among six olaparib-resistant Ph + lymphoid leukemia cell lines, KOPN55bi was the most sensitive to TKI, and we successfully obtained T315I and T315M sublines. Although we had tried to introduce T315I mutation into three other olaparib-resistant Ph + ALL cell lines, no TKI-resistant sublines were obtained [20]. Thus, we could not conclude whether poor response to the combination of ponatinib and asciminib is a common feature of Ph + lymphoid leukemia or not at this moment.

In conclusion, we confirmed higher levels of TKI resistance in the T315M-acquired sublines of three human Ph + leukemia cell lines, which were obtained via HR using the CRISPR/Cas9 system, in comparison with the T315I-acquired sublines. Thus, our human models of T315M-acquired Ph + myeloid leukemia cell lines first provided direct in vitro confirmation of the high levels of synergistic effects between reduced doses of ponatinib and standard therapeutic doses of asciminib.
